# Reactivity to Social Stress in Subclinical Social Anxiety: Emotional Experience, Cognitive Appraisals, Behavior, and Physiology

**DOI:** 10.3389/fpsyt.2016.00005

**Published:** 2016-01-27

**Authors:** Liviu G. Crişan, Romana Vulturar, Mircea Miclea, Andrei C. Miu

**Affiliations:** ^1^Department of Psychology, Babeş-Bolyai University, Cluj-Napoca, Romania; ^2^Department of Cell and Molecular Biology, “Iuliu Haţieganu” University of Medicine and Pharmacy, Cluj-Napoca, Romania

**Keywords:** social anxiety, Trier Social Stress Test, cortisol, speech anxiety behavior, cognitive biases

## Abstract

Recent research indicates that subclinical social anxiety is associated with dysfunctions at multiple psychological and biological levels, in a manner that seems reminiscent of social anxiety disorder (SAD). This study aimed to describe multidimensional responses to laboratory-induced social stress in an analog sample selected for social anxiety symptoms. State anxiety, cognitive biases related to negative social evaluation, speech anxiety behaviors, and cortisol reactivity were assessed in the Trier Social Stress Test (TSST). Results showed that social anxiety symptoms were associated with increased state anxiety, biased appraisals related to the probability and cost of negative social evaluations, behavioral changes in facial expression that were consistent with speech anxiety, and lower cortisol reactivity. In addition, multiple interrelations between responses in the TSST were found, with positive associations between subjective experience, cognitive appraisals, and observable behavior, as well as negative associations between each of the former two types of response and cortisol reactivity. These results show that in response to social stressors, subclinical social anxiety is associated with significant changes in emotional experience, cognitive appraisals, behaviors, and physiology that could parallel those previously found in SAD samples.

## Introduction

Social anxiety disorder (SAD) is one of the most common psychiatric disorders, with a lifetime prevalence of 6.7% in Europe (1) and 12.1% in the USA (2). SAD is associated with high individual and social burden related to poor social functioning and adjustment at work (3, 4), lower levels of academic and professional achievement (5), low quality of life (6), and high levels of comorbidity with other mental disorders (7).

Recent work indicated that subclinical or “subthreshold” social anxiety is also associated with significant individual burden. From a dimensional perspective (8), the severity of social anxiety symptoms can range from mild unpleasant experiences, such as increased emotional arousal and behavioral inhibition in social situations, to debilitating fear of negative evaluation, panic-like symptoms, and behavioral avoidance (9). Up to 20% of general population report subclinical levels of social anxiety symptoms, which can alter individual functioning in multiple life domains (10, 11) and quality of life (12).

Increased social anxiety is linked with dysfunctions at multiple levels [for review, see Ref. (13–15)]. Subjective experience during social interactions is characterized by high negative affect and low self-efficacy (16) or feelings of inferiority (17). At the cognitive level, social anxiety has been linked with increased self-focused attention (18) and negative interpretation biases (19) in social situations. Furthermore, both SAD and subthreshold social anxiety may involve altered biological reactivity to social stress. For instance, recent studies investigated hypothalamic–pituitary–adrenal (HPA) axis activity, a biological stress response system that may be dysregulated in anxiety disorders [for review, see Ref. (20)]. Considering that it is a risk factor for health problems [e.g., Ref. (21)], impaired HPA reactivity may also contribute to medical comorbidities of SAD (22).

Research on HPA activity and social anxiety produced divergent results, indicating increased (23, 24), decreased (25, 26), or similar levels of cortisol (27, 28) during social stress in high social anxiety compared to healthy control samples. It was recently emphasized that the divergence of findings may reflect differences in methodology and samples and that there is need for studies using standardized methods and data analysis (20). Indeed, social stress was induced in these studies using one or more tasks involving public speaking (24–28), mental arithmetic (23, 24, 26, 28), and short-term memory performance (23). Cortisol was assayed at various times relative to stress induction, either from saliva (24–26, 28) or plasma (23, 27). Samples included patients with SAD (23–25, 27, 28) or analog samples selected for subthreshold social anxiety [Study 2 in Ref. (25, 26)]. Finally, cortisol reactivity was assessed based on comparisons between baseline and stress levels (25, 28), difference scores (23), peak levels with baseline levels as covariate (27), or area under the curve for repeated measures (24, 26). In addition to this methodological heterogeneity, there is limited information on the links between cortisol levels and severity of social anxiety symptoms on the one hand, and subjective, cognitive, and behavioral responses to stress on the other hand. The available evidence suggests that trait shyness [Study 2 in Ref. (25)] and social anxiety symptoms (26) are associated with reduced cortisol reactivity, but that cortisol reactivity is positively associated with behavioral avoidance in SAD patients (24).

The present study investigated multidimensional responses to social stress in an analog sample selected for social anxiety symptoms. Social stress was induced using the Trier Social Stress Test (TSST) (29), a widely used standardized laboratory procedure that reliably triggers cortisol responses by combining elements of uncontrollability and social-evaluative threat (30). Considering that menstrual cycle phase and oral contraceptives use are known to influence cortisol reactivity in the TSST [(31), for review, see Ref. (32)], these variables were controlled for in this study. In addition to salivary cortisol, this study assessed subjective state anxiety, cognitive biases related to negative social evaluation, and speech anxiety behaviors during the TSST.

We used a correlational design to describe the associations between severity of social anxiety symptoms and subjective, cognitive, behavioral, and physiological responses to social stress. Previous correlational studies [e.g., Ref. (33, 34)] have shown that social anxiety is linked to an array of altered responses under stress, which warrants the use of a multidimensional approach in this field of research. Our study in subclinical anxiety explored new associations between HPA axis reactivity to stress and ratings of behavioral anxiety and cognitive biases that are central to SAD. Considering that social anxiety is a continuum from mild symptomatology to severe pathology (35–37), the present results in an analog sample with high social anxiety could be relevant for SAD as well.

## Materials and Methods

### Participants

**Figure 1:**
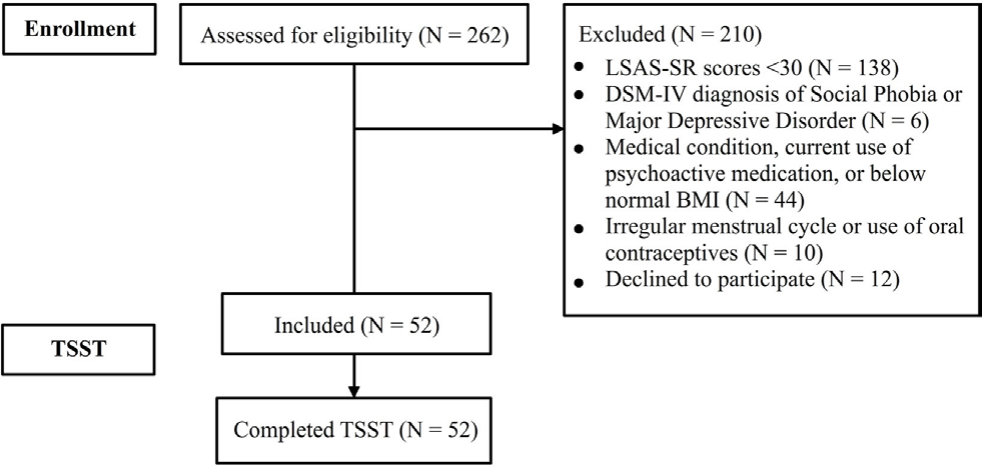
**Flow diagram describing the participant recruitment process for this study**. Abbreviations: BMI, body mass index; LSAS-SR, Liebowitz Social Anxiety Scale (self-report); TSST, Trier Social Stress Test.

A large pool of undergraduate students (*N* = 262) filled in the self-report version of the Liebowitz Social Anxiety Scale (LSAS-SR) (38). Exclusion criteria were (1) a score below 30 on LSAS-SR, indicating reduced levels of social anxiety symptoms (39); (2) meeting the clinical criteria for an anxiety or mood disorder, based on the Structured Clinical Interview for DSM-IV (40); (3) current diagnosis of endocrine, neurological, or psychiatric disorders, current use of psychoactive medication, and other medical characteristics (e.g., underweight body mass index) that may interfere with HPA functions (41); and (4) irregular menstrual cycle or use of oral contraceptives, which are known to influence cortisol reactivity in women (31). Therefore, only volunteers with LSAS-SR scores over 30, without anxiety and mood disorders, free of HPA-related medical conditions, and in the case of women, with regular menstrual cycle and who were not on medication relevant for HPA were recruited for this study (see Figure 1). The final sample consisted of *N* = 52 healthy participants (42 females; age: 19.96 ± 1.34 years), with increased social anxiety symptoms (LSAS-SR: *M* = 58.29, SD = 17.1; range 37–115). The study protocol complied with the ethical principles stated in the Declaration of Helsinki and was approved by the Ethics Committee of Babeş-Bolyai University. Participants signed an informed consent before entering the study.

### Social Anxiety Symptoms

The LSAS-SR (38) is a 24-item scale that quantifies fear and avoidance of social situations (e.g., giving a talk in front of an audience; taking a test). This is the self-report version of a clinician-administered scale (42), which has been widely used in clinical studies as a screening tool or outcome measure for SAD treatment. The overall score is most frequently used to summarize LSAS ratings, but other relevant subscales can also be derived (38). Two such subscales were also included in this study: the Total Fear scale (i.e., the sum of all fear ratings) and the Total Avoidance scale (i.e., the sum of all avoidance ratings). In line with previous reports (38), the reliability of LSAS-SR total score was excellent in this study (Cronbach’s alpha = 0.90). The two subscales had very good reliability indices as well (Cronbach’s alphas: Total Fear scale = 0.89 and Total Avoidance scale = 0.78).

### Trier Social Stress Test

Trier Social Stress Test sessions were scheduled in the afternoon to avoid the confounding effect of cortisol awakening response (41) and the following steep decline in cortisol levels (43). Participants refrained from alcohol, caffeine, and exercise at least 4 h before the TSST, as well as eating, drinking low pH soft drinks, and brushing their teeth at least 1 h before the TSST. To eliminate potential saliva contaminants, they rinsed their mouths with water immediately before the TSST. Because the menstrual cycle phase is known to affect HPA axis reactivity in the TSST, women reported the date of the last menstruation, the typical duration of a menstrual cycle, and whether menstrual cycles are regular. These participants were scheduled in the luteal phase (days 21–25) of their menstrual cycle, when cortisol reactivity to stress is relatively increased and similar to men’s (31).

A slightly modified version of the original TSST protocol (29) was used. Briefly, the procedure started with a 5-min baseline (i.e., −10 to −5 min relative to stress onset) during which participants sat in a comfortable position and quietly relaxed with eyes open. After the baseline, participants were instructed to take the next 5 min (i.e., −5 to 0 min) to prepare a speech for a simulated job interview that will be delivered in front of an evaluative committee of three experts and will also be videotaped for subsequent analyses of their performance. After the 5-min preparation period, the panel entered the room and participants gave the speech. After 5 min of free speech (i.e., 0 to +5 min), participants were requested to count backwards from 6233 in steps of 13 for another 5 min (i.e., +5 to +10 min). Participants were then debriefed by the experimenter and rested for 15 min (i.e., +10 to +25 min) and then for another 10 min (i.e., +25 to +35 min). Participants sat throughout the TSST and saliva samples for cortisol assays were obtained over a 40-min interval, at −5 (after baseline), 0 (after preparation), +10 (after stress induction), +25 (after 15 min of rest), and +35 min (after another 10 min of rest) with reference to the stress onset.

Because cortisol is known to increase in response to uncertainty and anxious anticipation, several studies pointed out that baseline cortisol levels could be contaminated unless an appropriate accommodation period is provided before the TSST (44). In our study, the participants were scheduled 30 min before the beginning of the TSST, during which they sat comfortably in the laboratory. They were briefed about the salivary cortisol measurements and were instructed to use the saliva collection devices. The participants then completed the baseline State-Trait Anxiety Inventory (STAI) (see below) and other questionnaires. Following this, the participants were left alone to rest comfortably for 5 min, and afterward they provided the first saliva sample (i.e., the baseline cortisol level at −5).

### State Anxiety

A 5-point Likert scale (0 = “not at all” and 4 = “very much”) was used to assess state anxiety throughout the TSST, in parallel with saliva collection for cortisol assay. In addition, state anxiety was also assessed immediately before baseline and immediately after stress induction in the TSST, using the state version of the STAI (45). The two measures were complementary in that the Likert scale could be repeatedly administered without significant delays in the TSST, whereas STAI offered a more detailed and reliable assessment (Cronbach’s alpha = 0.93 in this sample) before and after stress induction and facilitated comparison to other studies.

### Speech Anxiety Behaviors

Video recordings of participants’ speech performance in the TSST were independently assessed by three trained evaluators, using the Behavioral Assessment of Speech Anxiety (BASA) (46). BASA allows multidimensional assessments of speech anxiety based on six behavioral categories (i.e., voice, verbal fluency, mouth and throat, facial expression, arms and hands, and gross bodily movements). Each behavioral category contains one or more specific behaviors (e.g., behaviors included in the voice category: quivering or tense voice, talking too fast, talking too soft, and monotonous or lack of emphasis). In total, there are 17 specific behavioral manifestations that are rated on the BASA scale with an additional overall estimate of anxiety. Following the original BASA instructions, a total score of behavioral anxiety can also be computed by summing all the items. Each rater watched the video recordings and scored the BASA items on a 10-point scale that indicates the severity of anxiety (0 = “not at all” and 9 = “strong”), considering both the frequency and the intensity of a particular behavior. Thus, we obtained three independent ratings for each of the six behavioral categories and for the total anxiety score (see Table 1).

**Table 1:** **Anxiety behavior ratings based on the Behavioral Assessment of Speech Anxiety (BASA)**.

		Speech anxiety behaviors (BASA)						

		Voice	Verbal fluency	Mouth and throat	Facial expression	Arms and hands	Gross bodily movement	Total
Ratings	Rating #1	4.08 ± 0.39	10.66 ± 0.87	4.34 ± 0.38	10.79 ± 0.48	8.87 ± 0.59	1.72 ± 0.31	45.01 ± 2.27
	Rating #2	6.30 ± 0.53	13.12 ± 0.67	6.31 ± 0.45	12.97 ± 0.85	12.92 ± 0.87	1.76 ± 0.31	58.60 ± 2.91
	Rating #3	8.34 ± 0.64	17.60 ± 0.99	4.18 ± 0.39	13.46 ± 0.66	11.18 ± 0.59	1.24 ± 0.27	61.16 ± 2.41
	Average rating	6.24 ± 0.40	13.79 ± 0.63	4.94 ± 0.31	12.41 ± 0.57	10.99 ± 0.55	1.57 ± 0.23	54.92 ± 2.09

*Values in cells are means and SEMs. BASA contains 17 ratings of specific behaviors and one overall estimate of anxiety. Each item is rated on a 10-point severity scale (0 = not at all and 9 = strong). The BASA total score is the sum of all the items*.

### Cognitive Biases

Cognitive biases were assessed using the probability and cost of negative evaluation scale (47). Participants were asked to indicate the likelihood that their TSST performance will be negatively evaluated (e.g., “The raters will think you are incompetent”), and rate the consequences of such negative evaluations (e.g., “How bad would it be for you if the raters will think you are incompetent?”). In total, there were seven items rated on a 5-point scale (0 = “not at all” and 4 = “extreme”). Scale reliability in this sample was very good (Cronbach’s alpha = 0.88).

### Salivary Cortisol

Saliva samples were collected using standard collection devices (Salimetrics, CA, USA) and stored at −20°C until assaying. Salivary cortisol concentration was assessed by liquid chromatography–tandem mass spectrometry (48). The method is based on a chromatographic separation using a reverse-phase column; the eluate is routed into a triple-quadrupole mass spectrometer operating in the ion evaporation mode with an ion-spray ionization probe. The intra- and inter-assay coefficients of variation were 2.67 and 5.95%, respectively. Salivary cortisol was quantified in nanomoles per liter.

### Statistical Analyses

Repeated measures analysis of variance (ANOVA) and Student’s *t*-tests were used to investigate changes in salivary cortisol and state anxiety throughout the TSST. Associations between subjective, cognitive, behavioral, and physiological measures of anxiety were examined using Spearman’s correlations. For speech anxiety behaviors, agreement between the three independent evaluators was assessed based on intraclass correlations (ICC). ICC is suitable for more than two evaluators and incorporates the magnitude of the disagreement between evaluators, with larger magnitude disagreements resulting in lower ICC (49). Agreement is poor for ICC values <0.40, fair for values between 0.40 and 0.59, good for values between 0.60 and 0.74, and excellent for values between 0.75 and 1.0 (50). The five measurements of salivary cortisol included in the TSST were combined into an area under the curve index calculated with reference to the baseline (−5). This index, “area under the curve with respect to increase” (AUC_I_) was computed using the formula for repeated measurements with variable time between measurements presented in Pruessner et al (51). AUC_I_ emphasizes the changes over time in salivary cortisol, reflecting the sensitivity of HPA axis.

## Results

One participant was excluded from the analyses after inspecting the data for outliers. This participant had the lowest possible scores on all subjective anxiety measurements and on the cognitive biases scale. Another participant had missing salivary cortisol measurements due to insufficient saliva. Therefore, HPA-related analyses included only the remaining 50 participants.

### Self-Report State Anxiety

**Figure 2:**
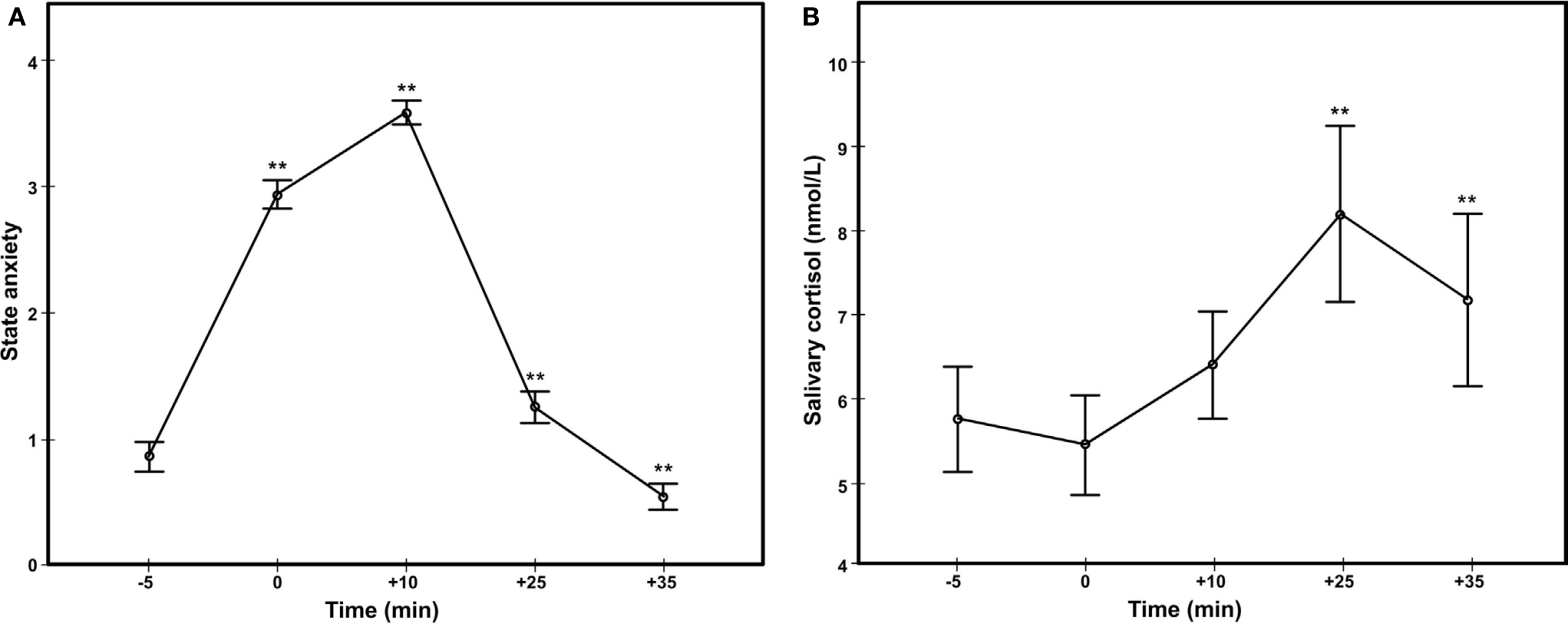
**Anxiety responses in the Trier Social Stress Test, based on Likert self-reported state anxiety (A) and salivary cortisol (B)**.

A repeated measures ANOVA was used to analyze the changes in state anxiety measured using Likert self-report scales during TSST. Because Mauchly’s test of sphericity was significant [χ(9) = 43.73, *p* < 0.001], the degrees of freedom were corrected using Greenhouse–Geisser method. A significant effect of time was found: *F*(2.77, 138.49) = 240.40, *p* < 0.001, $\eta_{P}^{2}=0.83$. *Post hoc* tests with Bonferroni correction showed a significant increase in state anxiety from baseline (−5) to anticipation (0) and from anticipation to stress (+10), and a significant decrease from stress to the two poststress assessments (+25 and +35) (Figure 2A).

A paired *t*-test was used to measure the effect of stress on the STAI ratings. There was a significant increase in state anxiety after stress (+10) compared to before stress (−10): *t*(50) = 18.39, *p* < 0.001, *d* = 2.57. As expected, Likert and STAI ratings of state anxiety after stress induction correlated significantly (*r*_s_ = 0.54, *p* < 0.001), so we used only the latter in all subsequent analyses.

Spearman’s rho was used to describe the relationship between LSAS and STAI ratings because a Shapiro–Wilk test revealed that LSAS scores did not meet normality assumptions. There was a significant correlation between severity of social anxiety symptoms and state anxiety after stress induction in the TSST (*r*_s_ = 0.25, *p* = 0.041).

### Cognitive Biases

Following stress induction in the TSST, cognitive biases related to the probability and cost of negative evaluation were assessed. The descriptive statistics for the two subscales are presented in Table 2. There were no significant differences between the ratings of probability (*M* = 17.25, SD = 5.58, range = 22) and cost biases (*M* = 16.71, SD = 4.79, range = 23) [*t*(50) = 0.77, *p* = 0.443].

**Table 2:** **Relations between social anxiety symptoms and cognitive biases of probability and costs of negative evaluation**.

	Variables	Descriptive statistics		Spearman’s correlations	
		Mean	SE	1	2
1	LSAS	58.20	2.42		
2	Probability biases	17.25	0.78	0.30^†^	
3	Cost biases	16.71	0.67	0.47**	0.56**

Values in cells are Spearman’s rho correlations with Bonferroni-adjusted alpha levels of 0.016 (^†^*p* < 0.05; **p < 0.001).

Spearman’s correlations with Bonferroni-adjusted alpha levels of 0.016 (0.05/3) revealed that the severity of social anxiety symptoms correlated positively with cognitive biases related to negative evaluation. Cost biases correlated positively with LSAS (*r*_s_ = 0.47, *p* = 0.001), while probability biases were marginally significant (*r*_s_ = 0.30, *p* = 0.018). As expected, there was a significant positive correlation between the two cognitive biases (*r*_s_ = 0.56, *p* < 0.001).

### Speech Anxiety Behaviors

Three independent ratings of speech anxiety behaviors were made using BASA, based on videos of participants’ speech performance in the TSST. The inter-rater reliability was excellent (average-measures ICC = 0.76), and an aggregate score of all three ratings was used in all further analyses. Table 3 shows speech anxiety behavior ratings for each specific domain and correlations with LSAS.

**Table 3:** **Relations between social anxiety symptoms and anxiety behavior ratings based on the Behavioral Assessment of Speech Anxiety (BASA)**.

	Variables	Descriptive statistics		Spearman’s correlations					
		Mean	SE	1	2	3	4	5	6
1	LSAS	58.20	2.42						
2	Voice	6.24	0.40	0.24^†^					
3	Verbal fluency	13.79	0.63	0.24^†^	0.30^†^				
4	Mouth and throat	4.94	0.31	0.02	0.57*	0.35^†^			
5	Facial expression	12.41	0.57	0.40*	0.40*	0.42*	0.34^†^		
6	Arms and hands	10.99	0.55	0.24^†^	0.33^†^	0.31^†^	0.28^†^	0.67*	
7	Gross bodily movement	1.57	0.23	−0.10	0.21	−0.06	0.15	0.18	0.27^†^

Values in cells are Spearman’s rho correlations with Bonferroni-adjusted alpha levels of 0.0023 (^†^*p* < 0.05; *p < 0.0023). 1: LSAS, Liebowitz Social Anxiety Scale (self-report); 2–7: BASA, Behavioral Assessment of Speech Anxiety.

Severity of social anxiety symptoms positively correlated with TSST speech anxiety behaviors in the domains of voice (*r*_s_ = 0.24, *p* = 0.048), verbal fluency (*r*_s_ = 0.24, *p* = 0.046), facial expression (*r*_s_ = 0.40, *p* = 0.002), and movement of arms and hands (*r*_s_ = 0.24, *p* = 0.048). After adjusting the alpha level with Bonferroni correction at 0.0023 (0.05/21), only the positive correlation between LSAS and BASA facial expression ratings remained significant.

### Cortisol Reactivity

A repeated measures ANOVA found significant variations of salivary cortisol during TSST [*F*(1.85, 88.77) = 5.65, *p* < 0.01, $\eta_{P}^{2}=0.11$]. Mauchly’s test of sphericity was significant [χ(9) = 98.93, *p* < 0.001]; therefore, the degrees of freedom were corrected using Greenhouse–Geisser method. *Post hoc* tests with Bonferroni corrections revealed a significant increase in salivary cortisol compared to baseline at 25 min after the stress onset (+25), followed by a significant drop in salivary cortisol at 35 min after the stress onset (+35) (Figure 2B).

**Figure 3:**
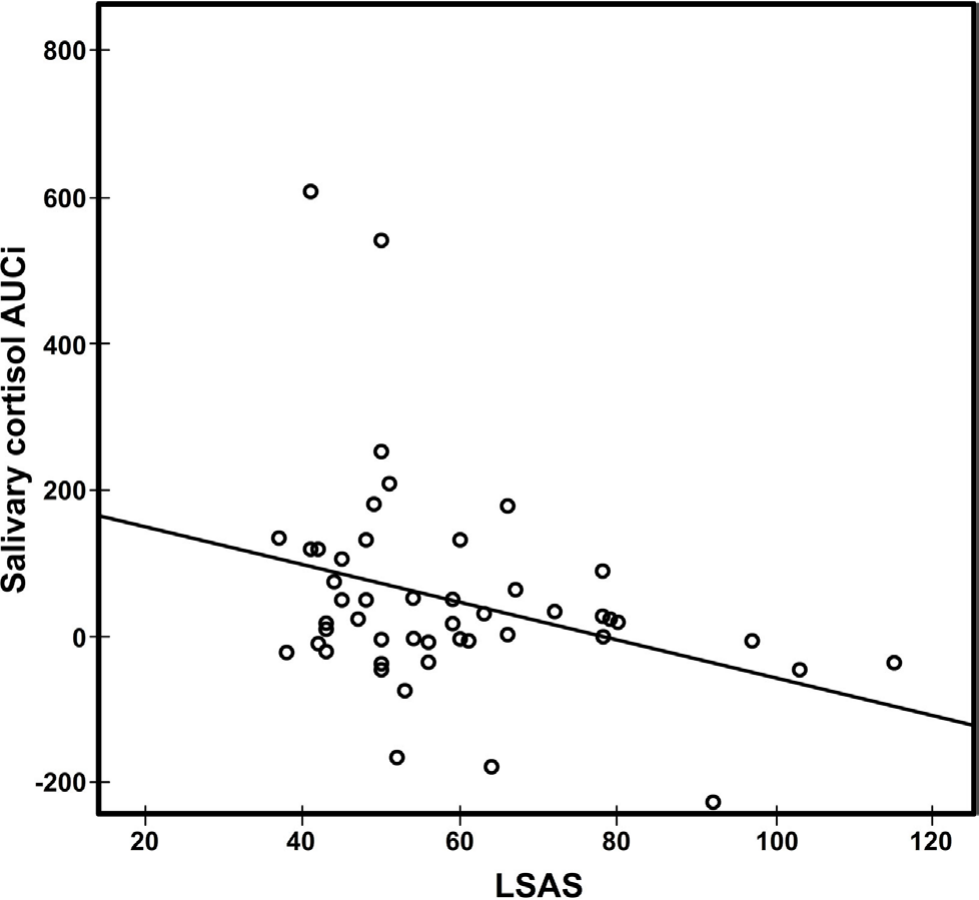
**Scatter plot of salivary cortisol AUC_I_ against LSAS scores (*r*_s_ = −0.29, *p* = 0.021)**. AUC_I_, area under the curve relative to increase; LSAS-SR, Liebowitz Social Anxiety Scale.

A Spearman’s rank-order correlation was used to determine the relationship between LSAS scores and cortisol AUC_I_, because Shapiro–Wilk test was significant for both variables. We found that the severity of social anxiety symptoms correlated negatively with cortisol AUC_I_ (*r*_s_ = −0.29, *p* = 0.021) in the TSST (Figure 3). Baseline cortisol levels did not correlate with STAI measure at baseline, cortisol AUC_I_, or LSAS (all *p* values >0.1).

### Relations between TSST Responses

**Figure 4:**
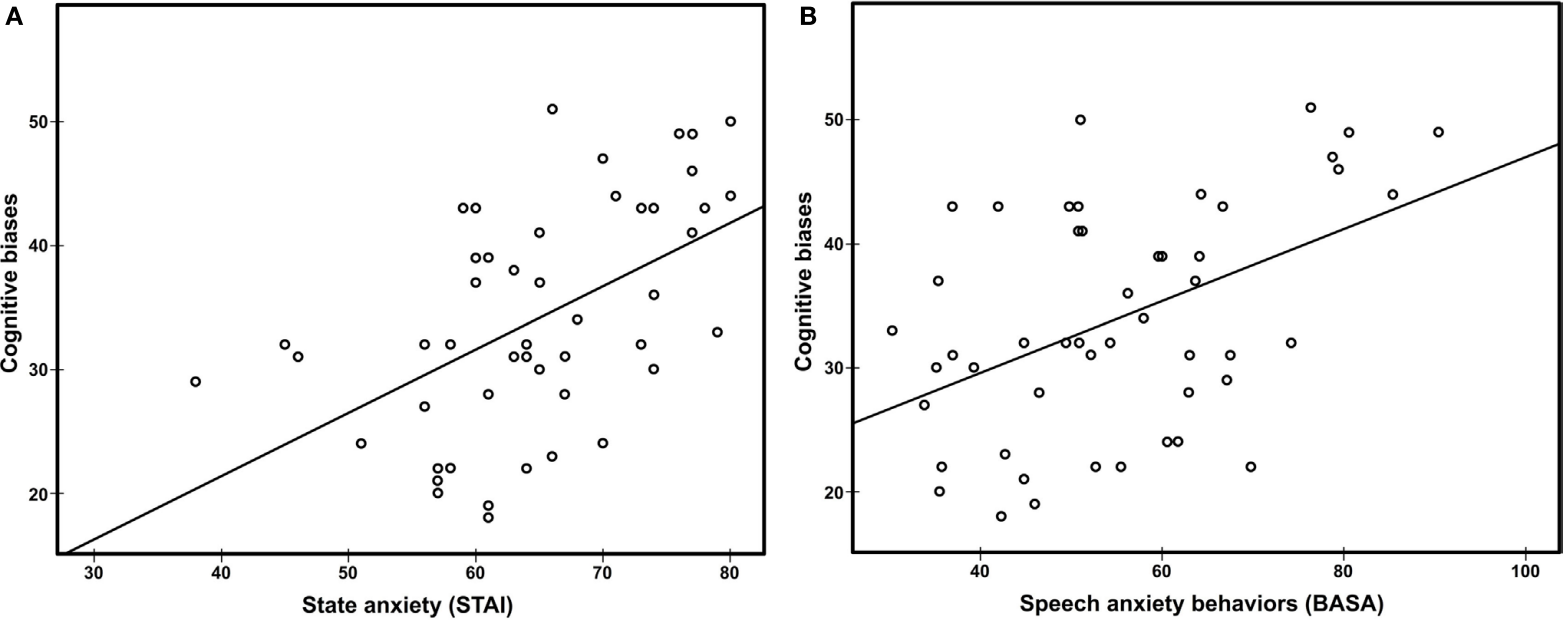
**Scatter plot of cognitive biases against state anxiety (A) *r*_s_ = 0.55, *p* < 0.008 and speech anxiety behaviors (B) *r*_s_ = 0.42, *p* < 0.008**. STAI, State-Trait Anxiety Inventory; BASA, Behavioral Assessment of Speech Anxiety.

Table 4 shows the correlations between STAI-state anxiety, speech anxiety behaviors, cognitive biases, and cortisol reactivity in the TSST. An overall score for speech anxiety behaviors and cognitive biases was used in these analyses. There was a positive correlation between state anxiety and cognitive biases related to negative evaluation (Figure 4A). Speech anxiety behaviors also correlated positively with cognitive biases (Figure 4B), but not with subjective state anxiety. These relationships remained significant after adjusting the alpha level at 0.0083 (0.05/6) using Bonferroni correction. In addition, state anxiety and cognitive biases, but not speech anxiety behaviors, correlated negatively with cortisol reactivity. These correlations were significant at the traditional alpha level but did not remain significant after correcting for multiple comparisons.

**Table 4:** **Relations between state anxiety, speech anxiety behaviors, cognitive biases related to negative evaluation, and salivary cortisol**.

	Variables	Descriptive statistics			Spearman’s correlations		
		Mean	SE		1	2	3
1	State anxiety (STAI)	64.61	1.29				
2	Speech anxiety behaviors (BASA)	54.92	2.09		0.20		
3	Cognitive biases	33.96	1.27		0.55*	0.42*	
4	Salivary cortisol AUC_I_	46.45	19.85		−0.33^†^	−0.04	−0.25^†^

Values in cells are Spearman’s rho correlations with Bonferroni-adjusted alpha levels of 0.008 (^†^*p* < 0.05; *p < 0.008). AUC_I_, area under the curve relative to increase; BASA, Behavioral Assessment of Speech Anxiety; STAI, State-Trait Anxiety Inventory.

### Supplementary Analyses

We ran a supplementary analysis on salivary cortisol. Since AUC_I_ is referenced to baseline, it is possible to get a negative value if the repeated measurements show a strong decrease over time (51). In our sample, 40% (*n* = 20) had negative AUC_I_ values, thus reflecting an overall decrease in salivary cortisol during TSST. We split the sample in two subgroups based on the participants’ AUC_I_ values (i.e., positive vs. negative), then compared the two subgroups on LSAS ratings. No significant differences were found on total LSAS scores. When analyzing the subscales, significant differences were found on LSAS Total Fear scores [*t*(48) = 2.11, *p* = 0.04, *d* = 0.58], but not on the Total Avoidance scores. In a *post hoc* analysis on the entire sample, we found a significant negative correlation between LSAS Total Fear and cortisol AUC_I_: *r*_s_ = −0.37, *p* = 0.004. This relationship was not found for the LSAS Total Avoidance scores.

When comparing the two subgroups on salivary cortisol measurements, no significant differences at any of the five time points were found. We also found no significant differences on baseline STAI ratings, but we did find a significant difference in STAI ratings during stress [*t*(48) = 2.14, *p* = 0.037, *d* = 0.63], in that the negative AUC_I_ group reported a higher level of state anxiety. Cost biases were also significantly higher in the negative AUC_I_ group [*t*(48) = 2.29, *p* = 0.027], but not probability biases.

## Discussion

The results of this study indicated that the severity of social anxiety symptoms was positively associated with self-reported state anxiety and biased appraisals related to negative social evaluation. Social anxiety symptoms also correlated positively with several observable anxiety behaviors in the TSST (i.e., voice, verbal fluency, facial expressions, and movements of arms and hands), but only the correlation with facial expression ratings remained significant after adjusting the alpha level for multiple comparisons. On the other hand, the severity of social anxiety symptoms was negatively associated with cortisol reactivity.

In addition, these results also uncovered links between multidimensional responses in the TSST, with positive interrelations between subjective experience and cognitive appraisals, as well between the latter and observable behaviors of speech anxiety. Negative relations between HPA reactivity and cognitive biases as well as state anxiety were also found, but they did not remain significant after adjusting the alpha level for multiple comparisons. Finally, in *post hoc* analyses, we identified a possible subtype of social anxiety, characterized by increased state anxiety and cost biases, but diminished HPA reactivity during stress.

Participants to this study were selected for high social anxiety on LSAS, a self-report scale that shows good sensitivity and specificity to diagnostic criteria for SAD. Clinical research reported that the cutoff score that was used in this study may identify over 93% of SAD patients (52). However, other studies in student samples (53) emphasized that LSAS scores over this cutoff may not necessarily indicate a diagnosis of SAD, but rather high social anxiety symptoms that are nonetheless associated with dysfunctions at multiple levels [for review, see Ref. (13–15)]. Indeed, these results showed that the severity of social anxiety symptoms is related to the magnitude of subjective, cognitive, behavioral, and physiological responses to social stress. Participants with higher scores on LSAS displayed increased state anxiety, biased appraisals related to the probability and cost of negative social evaluation, increased behavioral changes in facial expressions that were consistent with speech anxiety, and lower cortisol reactivity to social stress.

The finding of increased state anxiety and lower cortisol reactivity during social stress is in line with the results of a previous study in an analog sample selected for social anxiety symptoms (26). This pattern has also been observed in SAD, but it is unclear whether it is a general characteristic of this condition [Study 1 in Ref. (25)] or it is specific only to a subgroup of patients (24, 54). In our sample, we found that individuals with high social anxiety can show different patterns of HPA reactivity to acute social stress. Specifically, 40% of the participants showed an overall decrease in salivary cortisol during TSST. Interestingly, this group also had a higher level of state anxiety during stress and more severe symptoms of social anxiety on the LSAS Total Fear subscale. We are aware of a previous study that found a similar pattern in a clinical sample (24). The authors of that study compared salivary cortisol responses to TSST in patients with SAD, PTSD, and healthy controls. For a subgroup of SAD patients (39% of the sample), the distress was so high that the TSST procedure had to be adjusted (e.g., give verbal encouragements to the participants). Interestingly, these patients also displayed significantly lower salivary cortisol responses. In fact, only when controlling for this subgroup, significant differences between SAD patients and healthy controls could be found on salivary cortisol. Thus, although the authors of that study concluded that salivary cortisol is higher in SAD patients than in controls, this pattern may not characterize all SAD patients.

Reduced cortisol reactivity may be a relevant risk factor in socially anxious individuals. This characteristic was also found in other conditions, such as posttraumatic stress disorder, chronic fatigue syndrome, or fibromyalgia, and it was argued that it may be a transdiagnostic marker of chronic stress [for review, see Ref. (55)]. In the case of social anxiety, inability to adapt to social situations could in time result in allostatic load (56) and reduced cortisol reactivity, at least for a subgroup of individuals. This type of biological disengagement from social stress (57) may, in turn, contribute to persistence of social anxiety symptoms. For example, it could be the case that lower cortisol reactivity leads to an inappropriate energy mobilization in social situations, rendering socially anxious individuals unable to adapt and susceptible to poor performance. This, in turn, could reinforce cognitive biases of probability and costs of negative evaluation that sustain anticipatory anxiety and avoidance of social situations. Reduced cortisol reactivity could also increase comorbid medical problems (22, 58). More focused investigations on characterizing subgroups of individuals in both analog and clinical samples could lead to a better understanding of the specific vulnerabilities and treatment needs in social anxiety.

An alternative view of these results is that lower cortisol reactivity could reflect coping in the form of disengagement from social settings that involve the possibility of negative evaluations or social rejection (59, 60). This view is in line with a recently developed model of protective inhibition [protective inhibition of self-regulation and motivation (PRISM) (61)]. The PRISM model predicts that in social situations that induce hyperarousal or that allow for disengagement coping, social anxiety is related to decreased cortisol mobilization as part of a protective disengagement mechanism against unmanageable high emotional arousal. Indeed, the participants from our sample who were cortisol hyporesponders displayed higher scores on measures that indicate increased arousal: LSAS Total Fear, STAI during stress, and biases related to the cost of negative social evaluation. In contrast, their low cortisol levels were not related to measures that are less indicative of high arousal: LSAS Total Avoidance, baseline STAI, and biases in the probability of negative social evaluation.

Maladaptive responses to social situations may crucially involve biased appraisals related to the probability and cost of negative social evaluation. These biases have been associated with social anxiety symptoms, and there is evidence that they are specific to social events, they tend to be pervasive and may be involved as mechanisms of change in the response to cognitive-behavioral psychotherapy for social anxiety [for review, see Ref. (62)]. In addition to replicating their association with social anxiety symptoms, this study also shows that the probability and cost biases are related to lower cortisol reactivity to social stress. Specifically, cost biases were significantly higher in the subgroup of participants who had reduced cortisol response to stress. Future studies may try to manipulate these biases in order to test their causal involvement in cortisol reactivity. In two clinical trials on cognitive-behavioral therapy with SAD patients, cost biases were shown to mediate treatment outcomes and could thus predict long-term change in symptomatology (63, 64). If evidence will emerge that bias modification also restores cortisol reactivity to social stress, this intervention may provide an effective way to reduce both current social anxiety symptoms and biological dysfunctions that may contribute to later symptom reinstatement and comorbid health risks. There are several methods that could prove to be effective, such as cognitive restructuring and mindfulness techniques (65) or computerized cognitive bias modification procedures (66, 67).

It was recently emphasized that studies on HPA reactivity in anxiety need to implement standardized methods and data analysis in order to increase finding reliability (20). This study used TSST, a standardized laboratory procedure that reliably induces social stress and HPA reactivity (29, 30). Considering the significant influence of menstrual cycle phase and contraceptive use on cortisol reactivity in the TSST [(31), for review, see Ref. (32)], these potential confounds were controlled in this study. In addition, this study employed a widely used reactivity formula for repeated measures cortisol assays (i.e., AUC_I_) (51). All these efforts may have contributed to the lineup of positive findings, across experience, cognition, behavior, and physiology. However, the relatively small sample size and unequal sex distribution may limit the generality of these findings. Furthermore, the lack of a control group limits our conclusions regarding the significance of the negative correlation between severity of social anxiety symptoms and cortisol reactivity. Since our sample consisted only of healthy individuals selected for high social anxiety, it remains unclear whether the same relationship can be found in low social anxiety individuals or in SAD patients. However, from a dimensional perspective of social anxiety (37), it is reasonable to expect similar results at both ends of the continuum. Future studies may try to clarify this aspect by recruiting participants with a wider range of social anxiety severity, including SAD patients. Another limit we must acknowledge is that the finding of a subtype of social anxiety with blunted cortisol responses is based on *post hoc* analyses and should thus be interpreted with caution.

In conclusion, this study showed that symptom severity is associated with differences in social stress experience, cognitive appraisals, anxiety behavior, and HPA reactivity in an analog sample selected for social anxiety. Considering that these multidimensional characteristics are reminiscent of SAD, these results highlight current dimensional approaches to social anxiety.

## Conflict of Interest Statement

The authors declare that the research was conducted in the absence of any commercial or financial relationships that could be construed as a potential conflict of interest.
